# CDR3 Variants of the TXB2 Shuttle with Increased TfR1 Association Rate and Enhanced Brain Penetration

**DOI:** 10.3390/pharmaceutics15030739

**Published:** 2023-02-23

**Authors:** Pawel Stocki, Jaroslaw Szary, Mykhaylo Demydchuk, Leandra Northall, Charlotte L. M. Rasmussen, Diana Bahu Logan, Aziz Gauhar, Laura Thei, Shu-Fen Coker, Torben Moos, Frank S. Walsh, J. Lynn Rutkowski

**Affiliations:** 1 Ossianix, Inc., Stevenage Bioscience Catalyst, Gunnels Wood Rd., Stevenage SG1 2FX, UK; 2 Laboratory of Neurobiology, Department of Health Science and Technology, Aalborg University, 9220 Aalborg, Denmark

**Keywords:** blood–brain barrier (BBB), transferrin receptor 1 (TfR1), transcytosis, brain delivery, variable domain of new antigen receptor (VNAR)

## Abstract

Since the delivery of biologic drugs to the brain is greatly hampered by the existence of the blood–brain barrier (BBB), brain shuttles are being developed to enhance therapeutic efficacy. As we have previously shown, efficient and selective brain delivery was achieved with TXB2, a cross-species reactive, anti-TfR1 VNAR antibody. To further explore the limits of brain penetration, we conducted restricted randomization of the CDR3 loop, followed by phage display to identify improved TXB2 variants. The variants were screened for brain penetration in mice using a 25 nmol/kg (1.875 mg/kg) dose and a single 18 h timepoint. A higher kinetic association rate to TfR1 correlated with improved brain penetration in vivo. The most potent variant, TXB4, showed a 3.6-fold improvement over TXB2, which had on average 14-fold higher brain levels when compared to an isotype control. Like TXB2, TXB4 retained brain specificity with parenchymal penetration and no accumulation in other organs. When fused with a neurotensin (NT) payload, it led to a rapid drop in body temperature upon transport across the BBB. We also showed that fusion of TXB4 to four therapeutic antibodies (anti-CD20, anti-EGFRvIII, anti-PD-L1 and anti-BACE1) improved their brain exposure between 14- to 30-fold. In summary, we enhanced the potency of parental TXB2 brain shuttle and gained a critical mechanistic understanding of brain delivery mediated by the VNAR anti-TfR1 antibody.

## 1. Introduction

The blood–brain barrier (BBB) is formed of a tightly packed layer of endothelial cells that together with pericytes and astrocytes separate the blood stream from the brain. It is a selective barrier preventing unwanted pathogens from free penetration to the brain parenchyma while allowing active transport of nutrients critical for maintaining cellular function. The presence of the BBB also restricts large therapeutic molecules such as antibodies or enzymes from entering the brain, thus significantly reducing their clinical efficacy.

While many different approaches have been proposed to tackle the brain delivery of large therapeutic payloads, receptor mediated transcytosis (RMT) using antibodies against BBB receptors remains a widely studied non-invasive method. Alternative approaches were proposed including the use of nanoparticles with BBB targeting components [1,2]. Several receptors have been reported as suitable for targeted brain delivery of therapeutics, TfR1 remains one of the most well described and potent facilitators of RMT. Despite observations that brain pathologies can affect BBB composition and its protein expression profile [3], continuous expression of TfR1 in brain capillaries was observed regardless of the age [4] and remained unchanged in Alzheimer’s disease (AD) [5].

Significant advances were made with anti-TfR1 antibodies as brain shuttles for delivering of iduronate-2-sulfatase (IDS) for mucopolysaccharidosis type II (MPSII), anti-amyloid beta antibody (gantenerumab) for AD and progranulin for frontotemporal dementia, with clinical trials ongoing [6,7,8,9]. These shuttles take contrasting approaches with either a high or low affinity TfR1 antibody module, in either a mono- or bi-valent format, and in either a N- or C-terminal orientation [10,11,12]. Despite some observations that low affinity, mono-valent anti-TfR1 antibodies were superior to high affinity antibodies for effective brain delivery of therapeutic payloads [13], when compared side-by-side (and corrected for molecular weight) for IDS delivery the two contrasting shuttle systems presented a similar pharmacodynamic effect in vivo despite up to 1000-fold difference in affinity to TfR1 [14].

Effects of affinity, valency and orientation described for monoclonal antibodies to TfR1 have not been observed with brain shuttles based on VNAR antibodies to TfR1, such as TXB2 [15]. Single domain VNAR antibodies have a distinctively different architecture, allowing them to bind epitopes inaccessible to conventional IgG-based antibodies [16,17]. A unique binding mode and epitope recognition is most likely responsible for the distinctive characteristics of TXB2 brain shuttle. TXB2 showed high affinity to TfR1, cross-species reactivity, efficient parenchymal transcytosis across BBB without potential safety concerns related to target depletion or clearance of TfR1-expressing reticulocytes. In combination with brain specificity, low blood clearance and extended half-life resulting in high and sustained brain exposure, TXB2 presents an attractive shuttle for therapeutic delivery.

Here, we modified the CDR3 loop of the TXB2 brain shuttle with restricted randomization and identified variants with enhanced brain penetration in mice, which correlated with increased association rate to recombinant mouse TfR1 in vitro. The greatest improvement in brain penetration was observed for TXB4, which showed a 3.6-fold improvement over the parental TXB2 as a VNAR-Fc fusion protein. When fused to a series of therapeutic antibodies, TXB4 retained nearly a 3-fold improvement over TXB2 and enhanced brain penetration by 14- to 30-fold in comparison to unmodified antibodies. Like TXB2, TXB4 readily crossed the BBB and was detected in brain capillaries, parenchyma and neurons, but did not accumulate in peripheral organs.

## 2. Materials and Methods

**Figure 1:**
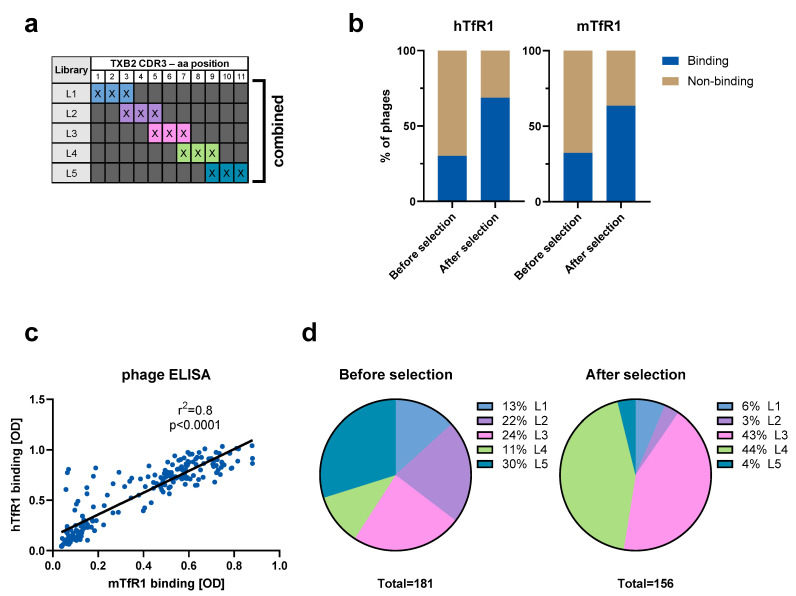
TXB2 maturation library design and selection. (**a**) Schematics of the CDR3 region of TXB2 antibody randomized in segments of three residues (X—any amino acid) per sub-library. Total of five sub-libraries L1–L5 were prepared individually before combining them and using for the selection against hTfR1. (**b**) Phage ELISA results presented as the percentage of binders to hTfR1 and mTfR1 from randomly picked clones before and after selection (round 1 output). (**c**) Pearson’s correlation analysis of phage ELISA-based binding of selected clones to hTfR1 and mTfR1. (**d**) Distribution of CDR3 sub-libraries in randomly picked clones from before and after selection.

**Phage selections.** Phage maturation libraries with restricted randomization of the CDR3 region of the parental TXB2 VNAR antibody were prepared by GeneWiz. Three residues per sub-library were randomized using degenerate NNK codons. Five sub-libraries were prepared in total as depicted in Figure 1a. The five sub-libraries were combined and used for one round of selection against recombinant human TfR1 (hTfR1) ectodomain as described before [15].

**Phage ELISA.** The method followed the protocol as previously described [15]. In brief, individual clones from before (input) and after (output) selection were picked. Supernatants from helper phage infected liquid cultures were recovered and used for ELISA against hTfR1 and recombinant mouse TfR1 (mTfR1) ectodomains (Sino Biological, Beijing, China).

**Production of fusion protein constructs.** VNAR-hFc formats were produced with VNARs at N-terminal end of hFc IgG1 with attenuated effector function (AEF) [18]. Neurotensin (NT) constructs were designed to include the peptide on the C-terminal end of hFc following a 3xG4S linker. Bivalent N-terminal heavy chain VNAR-antibody fusions (HC2N) were prepared using 3xG4S linkers with antibodies to CD20 (rituximab) [19], EGFRvIII [20], BACE-1 and PD-L1 (durvalumab) [21]. VNAR-hFc formats and NT constructs were expressed using Expi293F system (Thermo Fisher, Waltham, MA, USA), and antibody fusions were expressed in CHO cells. Proteins were affinity purified using a HiTrap MabSelect SuRe column (GE Healthcare, Chicago, IL, USA) followed buffer exchange to PBS, pH 7.4 using a HiPrep 26/10 Desalting column (GE Healthcare) as previously described [15].

**Tissue ELISA.** Female BalB/C mice (6–12 weeks old) received a tail vein injection at a dose of 25 nmol/kg. Tissue samples were collected 18 h post injection following cardiac perfusion with 25 mL of PBS supplemented with 1 EU/mL of heparin. Sample preparation and the ELISA protocol followed the methods described before [15]. In brief, milk-blocked supernatants recovered from homogenized tissues or plasma were analyzed by ELISA using anti-hFc capture and detection antibodies. The absolute concentrations were determined from standard curves prepared individually for each fusion protein using 4-parametric regression analysis.

**Binding kinetics.** Binding kinetics of fusion proteins were determined by surface plasmon resonance (SPR) using a Biacore T200 (GE Healthcare) as described before [15]. In brief, a His-capture kit (GE Healthcare) was used to immobilize His-tagged mTfR1 or hTfR1 in 0.1% BSA in HBS-EP+ buffer (GE Healthcare) on CM5 chip. Capture levels for mTfR1 and hTfR1 were approximately 90 and 250 RU, respectively. Analyte binding was measured using the single cycle kinetic SPR method. Analytes were injected at increasing concentrations (0.98, 3.9, 15.6, 62.5 and 250 nM) in HBS-EP+ at flow rate 30 μL/min. A flow cell without TfR1 captured served as a reference. Sensorgrams were fitted using 1:1 binding model and kinetic constants were determined using Biacore T200 evaluation software (GE Healthcare).

**Body temperature measurements.** Four mice per group received tail vein injections with either TXB4-NT fusions or control hFc-NT at the dose of 25 nmol/kg IV and body temperature was monitored over a 24 h period using subcutaneous transponders and DAS-8007 reader system (Plexx, Elst, The Netherlands).

**Immunohistochemistry (IHC).** Brain penetration in vivo was assessed by IHC using female BalB/C mice as described before [15]. In brief, mice received a single tail vein injection of the test antibodies at 25 nmol/kg (PD-L1—3.65 mg/kg; TXB4-PD-L1—4.32 mg/kg). After 18 h exposure, mice were deeply anesthetized and perfused. Serial coronal brain sections (40 μm) were cut, blocked and incubated overnight with goat anti-human IgG antibody (BA 3000, Vector Laboratories, Burlingame, CA, USA) diluted 1:200 in blocking buffer. After 24 h, the sections were washed and visualized using the Avidin–Biotin Complex-system (Vectastain ABC kit, Vector Laboratories). All images were captured on a brightfield Zeiss Axioskop microscope at 20× magnification with the same acquisition parameters.

## 3. Results

The TXB2 single domain VNAR antibody against TfR1 was the most potent hit identified from our semi-synthetic VNAR libraries using in vitro/in vivo phage display-based discovery process [15]. For affinity maturation, a phage library was prepared using TXB2 scaffold with uniquely randomized CDR3 region. A total of five sub-libraries (L1–L5) with three successive randomized residues were prepared (Figure 1a). Each consecutive sub-library contained one residue overlap with the previous library and a total of 11 residues were randomized in the CDR3 region.

The five sub-libraries were combined and used for selection against hTfR1. After 1 round of selection, the percentage of hTfR1 and mTfR1 binding clones in libraries increased from 30% to 68%, and 32% to 64%, respectively, as shown by phage ELISA (Figure 1b). The species cross-reactivity of TXB2 [15] was retained in the variants showing strong correlation in binding between hTfR1 and mTfR1 (Figure 1c). Sequencing representative clones picked before (181 in total) and after selection (156 in total) showed that the sub-library distribution before selection was nearly equal, while the distribution after the selection was dominated by the L3 and L4 libraries (Figure 1d). The TXB2 variants enriched after selection against hTfR1 had residue substitutions predominantly in position 5–9 of the CDR3 region. Very few substitutions were found in the flanking regions, in positions 1–4 (L1 and L2) and 10, 11 (L5).

**Figure 2:**
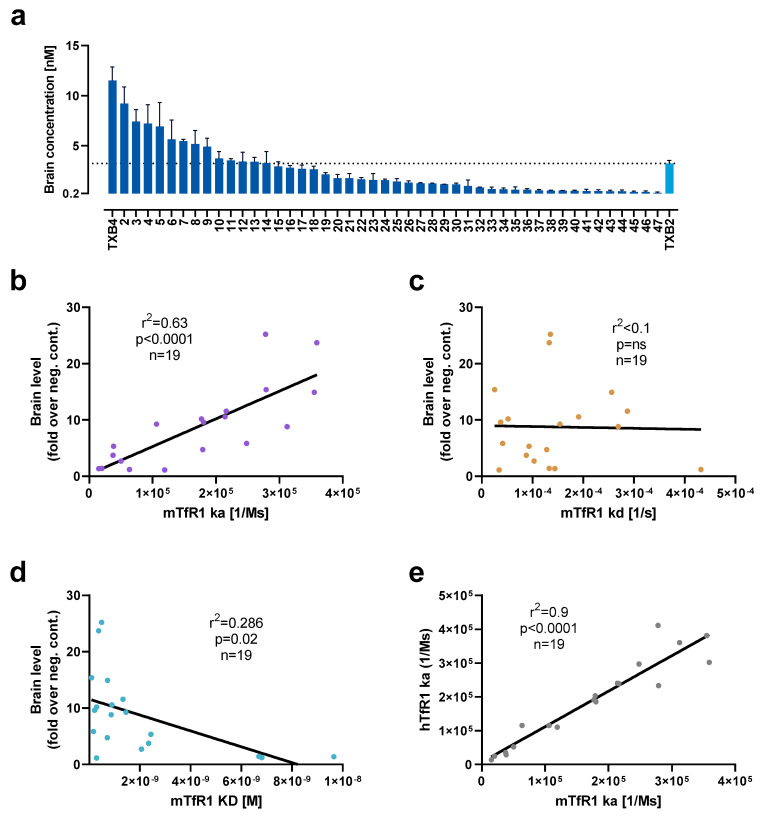
Functional activity of TXB2 variants in vivo correlates with TfR1 association rate in vitro. (**a**) A panel of 47 TXB2 variants were tested for brain penetration in mice at a dose of 25 nmol/kg (1.875 mg/kg), IV, and brain levels were measured at 18 h timepoint via ELISA (mean ± SD, *n* = 3). Levels of parental TXB2 and a negative isotype control (0.2 nM) at the same dose were used as references. TfR1 binding kinetics of 19 TXB2 variants selected for either low and high brain levels were assessed via SPR. (**b**) Association rate (ka), (**c**) dissociation rate (kd) and (**d**) affinity (KD) were independently correlated using Pearson’s method with the brain levels normalized to fold over negative control (neg. cont.). (**e**) Pearson’s correlation of the ka between hTfR1 and mTfR1 for the 19 TXB2 variants.

CDR3 variants of TXB2 were synthesized, cloned into a mammalian vector, and expressed as bivalent VNAR-hFc fusions by transient transfection in Expi293F cells. After TfR1 binding was confirmed in crude extracts, 47 TXB2 variants were expressed at medium scale and purified via affinity chromatography. To assess brain penetration, mice were administered 25 nmol/kg (1.875 mg/kg) by tail vein injection and brains were collected after 18 h following cardiac perfusion. A continuum of brain penetration was observed with 18 CDR3 variants reaching brain levels equivalent to or greater than parental TXB2 while the remainder were below or near the 0.2 nM level of the negative control (Figure 2a). In addition, 6 TXB2 variants that lost TfR1 binding were tested, and no brain penetration above negative control was observed (data not shown). The most potent variant, TXB4, reached a brain concentration of 11.5 nM at 18 h timepoint, which represented a 3.6-fold increase relative to the parental TXB2.

To further investigate the observed variability in brain penetration, 19 unique TXB2 variants with either high or low brain levels were selected for detailed evaluation of their TfR1 binding kinetics by SPR. There was a significant, strong, positive correlation for association rate of TXB2 variants for mTfR1 and brain penetration in mice (r^2^ = 0.63, *p* < 0.0001, *n* = 19) (Figure 2b). No correlation was detected for dissociation rate (Figure 2c), and a positive trend was observed for affinity (calculated as ratio of dissociation to association rate) and brain penetration (Figure 2d). There was also a significant, very strong, positive correlation between the ka for hTfR1 and mTfR1 (r^2^ = 0.9, *p* < 0.0001, *n* = 19) indicating high cross-species reproducibility of the binding kinetic results for TXB2 variants (Figure 2e).

The comparison of binding kinetic parameters of TXB2 and TXB4 showed marginally higher affinity of TXB4 (KD = 0.55 nM) in comparison to TXB2 (KD = 0.65 nM) to mTfR1. However, despite similar affinity, TXB4 had a 2.1-fold faster association rate and 1.8-fold faster dissociation rate in comparison to TXB2 (Table 1). The trend was similar for hTfR1, where the overall affinity of TXB4 (KD = 3.4 nM) was higher by 2.1-fold in comparison to TXB2 (KD = 7.2 nM), with increased association and dissociation rates by 3- and 1.4-fold for TXB4 over TXB2, respectively (Table 1).

**Figure 3:**
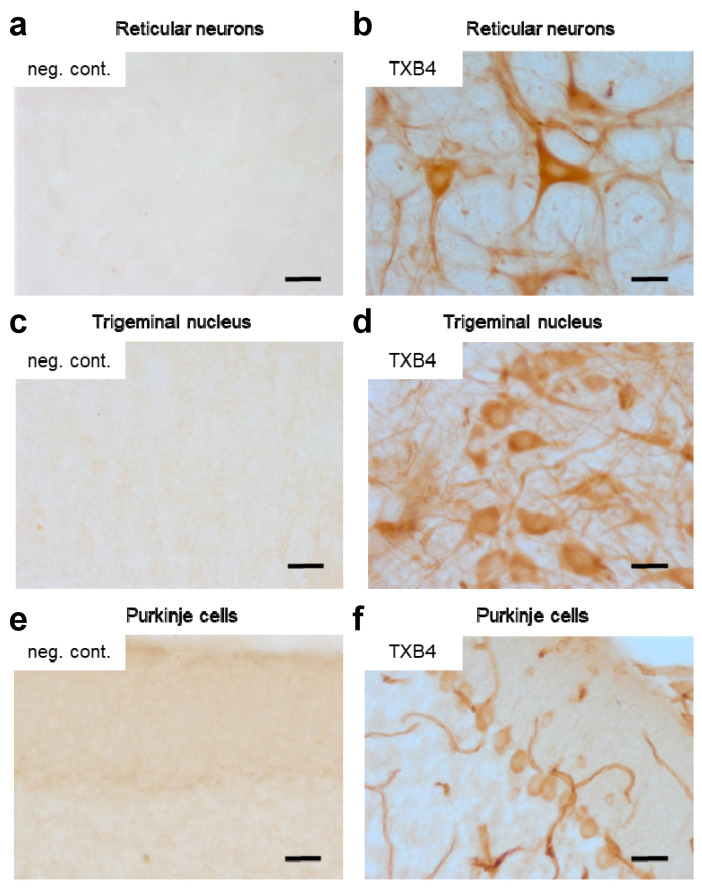
IHC assessment of brain penetration by the TXB4 shuttle. Mice were dosed with either TXB4 or negative control VNAR-hFc (neg. cont.) at 25 nmol/kg (1.875 mg/kg), IV and brains were collected following cardiac perfusion 18 h post injection (*n* = 2). Brain sections were analyzed for the presence of exogenous antibodies by IHC and images were collected from representative regions for comparison. Sections taken from the thalamus showed the distribution of (**a**) the negative control versus (**b**) TXB4 with localization in reticular neurons. Pronounced TXB4 staining was seen in trigeminal neurons in the midbrain (**d**) and Purkinje cells in cerebellum (**f**), where diffused parenchymal staining and BCEC staining were clearly distinguishable; the negative control showed no staining in corresponding sections (**c**,**e**). Scale bars at 50 μm.

To determine if the higher brain levels seen with TXB4 resulted from enhanced BBB transport and not from accumulation in brain capillaries, the localization of TXB4 within the mouse brain was examined via IHC. At 18 h after a single IV dose at 25 nmol/kg, TXB4 immunoreactivity was observed both diffusely and in specific cellular elements including brain capillary endothelial cells (BCECs), choroid plexus epithelial cells, and in neurons in all brain areas. BCEC staining was seen throughout the entire CNS, but the intensity did not exceed that found in many neuronal populations. Neurons exhibiting TXB4 immunoreactivity were distributed widely in different nuclei and brain regions with the most prominent staining in the lower brain stem (Figure 3), especially reticular neurons of the thalamus (Figure 3b), trigeminal neurons in the medulla oblongata (Figure 3d) and Purkinje cells in the cerebellum (Figure 3f). The VNAR-Fc negative control showed immunoreactivity only in epithelial cells of the choroid plexus (Supplementary Figure S1), without labeling of BCECs or neurons as shown in the respective control sections (Figure 3a,c,e).

**Figure 4:**
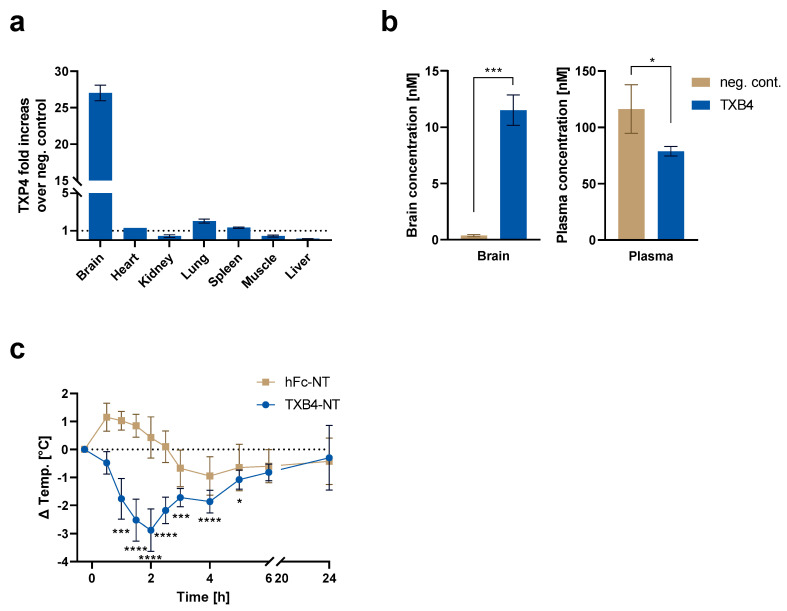
Brain-specific biodistribution of TXB4 and pharmacodynamic activity of TXB4 fused to NT. Mice were dosed with TXB4 or negative isotype control (neg. cont.) at 25 nmol/kg and blood and various organs were collected 18 h after cardiac perfusion. (**a**) Organ concentrations were measured via ELISA and were presented as fold increase over negative control (mean ± SD, *n* = 2). (**b**) Brain and plasma concentrations were assessed via ELISA (mean ± SD, *n* = 3) and significance was determined via two-tailed, unpaired *t*-test with * *p* < 0.05, *** *p* < 0.0002. (**c**) The TXB4-NT fusion or NT fused to the Fc domain alone (hFc-NT) were administered to mice at 25 nmol/kg and body temperature was continuously monitored for 24 h. Data presented as a change (Δ) from the pre-dose body temperature measured 15 min before dosing (mean ± SD, *n* = 4.). Significance was determined via two-way ANOVA and Dunnett’s comparison with * *p* < 0.05, *** *p* < 0.0002 and **** *p* < 0.0001.

It was previously shown that TXB2 exhibited a very high degree of brain specificity without accumulation in other organs, which also express TfR1 [15]. To determine if this feature was retained by TXB4, organ biodistribution was assessed in mice 18 h post IV dosing at 25 nmol/kg and compared to the negative isotype control. As seen with TXB2, there was no accumulation of TXB4 in any of the tested organs except for the brain where TXB4 showed 27-fold increase over control (Figure 4a). The reduced concentrations were observed mainly in liver but also in kidney and muscle (Figure 4a). The reduction might have resulted from reduced plasma levels of TXB4, which were 1.5-fold below negative control, which in itself could be attributed to potent and selective targeting to the brain (Figure 4a,b).

NT is a 13 amino acid neuropeptide that regulates physiological responses such as body temperature from within the CNS [22]. To confirm the ability of TXB4 to cross the BBB and trigger a physiological response, NT was fused to the C-terminus of the Fc domain (TXB4-NT). Body temperature was continuously measured for 24 h in mice administered with TXB4-NT at 25 nmol/kg, IV. There was a rapid reduction in temperature with maximal −2.9 °C drop at the 2 h timepoint (Figure 4c). The recovery from hypothermia from the 2 h timepoint onwards was consistent with the receptor desensitization and proteolytic cleavage of the NT peptide [23]. The NT peptide fused to the C-terminal of the hFc domain alone (hFc-NT) was used as a control and when dosed at the same concentration it failed to elicit a physiological response.

**Figure 5:**
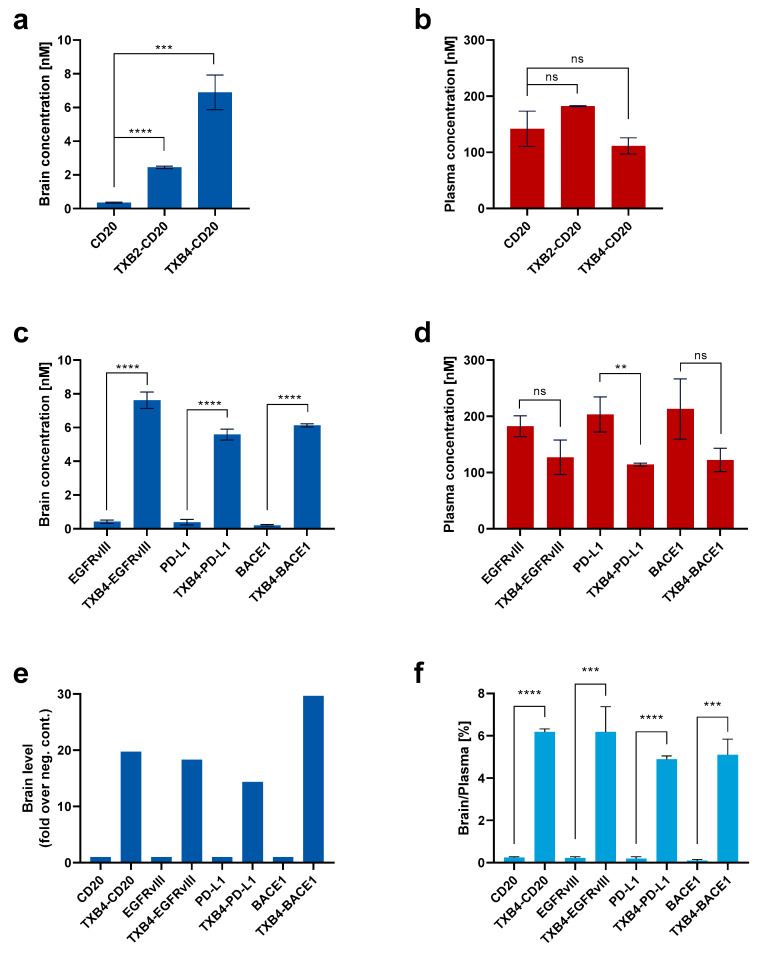
TXB4 is highly efficient in therapeutic antibody delivery to the brain. Antibody to CD20 alone, TXB2-CD20 and TXB4-CD20 fusions were dosed in mice at 25 nmol/kg. At 18 h post injection (**a**) brains and (**b**) plasma were collected and analyzed by ELISA (mean ± SD, *n* = 3). Therapeutic antibodies to EGFRvIII, PD-L1 and BACE1 as unmodified or as TXB4 fusions were dosed in mice at 25 nmol/kg with (**c**) brains and (**d**) plasma collections performed at the 18 h timepoint post injection followed by ELISA analysis (mean ± SD, *n* = 3). (**e**) Brain penetration data for therapeutic antibodies (compiled from Figure 5a,c) presented as average fold over corresponding unmodified antibody (fold increase over control). (**f**) Percentage brain/plasma ratio data for therapeutic antibodies (compiled from Figure 5a–d) (mean ± SD, *n* = 3). Significance at ** *p* < 0.01, *** *p* < 0.005, **** *p* < 0.0001 using two-tailed, unpaired *t*-test, and ns indicates not significant difference.

The CD20 antibody was used to compare the efficiency of the TXB4 in transporting a therapeutic biologic relative to the original TXB2. The VNARs were fused to the N-terminal end of the antibody heavy chain to produce a bivalent, bispecific TfR1/CD20 antibody. Mice were dosed at 25 nmol/kg, IV, and plasma and brain tissues were isolated following cardiac perfusion. Fusion to either shuttle resulted in a robust and significant increase in the CD20 antibody brain concentration 18 h post-injection and TXB4 was approximately 3-fold more potent than the original TXB2 (Figure 5a). Plasma levels for CD20 antibody and TXB2-CD20 fusion were comparable (Figure 5b), and while insignificant reduction was observed for TXB4-CD20 fusion, the trend could be related to a greater brain penetration. Evaluation of the TXB4 shuttle was extended to include antibodies to EGFRvIII, PD-L1 and BACE1. TXB4-antibody fusions were produced in the same format and mice were treated in the identical manner. Robust increases in brain concentration (ranging from 5.6 nM to 7.6 nM) were observed with the three additional bispecifics compared to the unmodified antibodies with levels similar to those seen with the CD20 fusion (Figure 5c). As with the TXB4-CD20 fusion, plasma concentration showed a trend toward reduction at the 18 h timepoint, but statistical significance was only reached for TXB4-PD-L1 (Figure 5d). When represented as fold increase over the unmodified antibody, fusion with the TXB4 shuttle resulted in 20-, 18-, 14- and 30-fold improvement in brain penetration for the CD20, EGFRvIII, PD-L1 and BACE1 antibodies, respectively (Figure 5e). When percentage brain to plasma ratios were calculated, the unmodified antibodies averaged 0.19%, but increased on average to 5.6% when fused to the brain shuttle TXB4 (Figure 5f).

**Figure 6:**
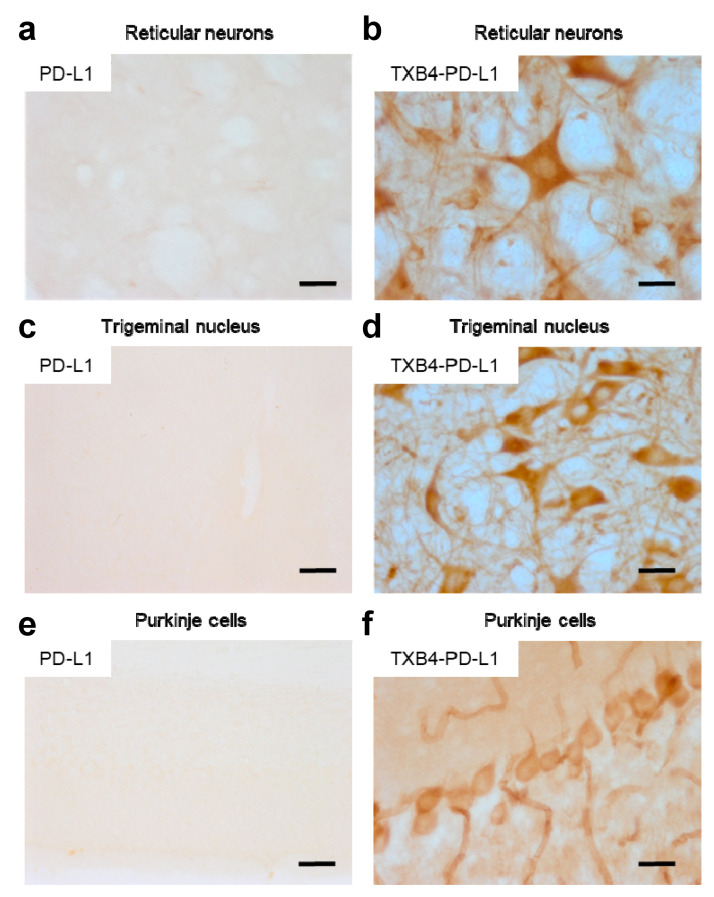
IHC assessment of antibody delivery to the brain parenchyma using the TXB4 shuttle. Mice were dosed with either the TXB4-PD-L1 fusion or unmodified antibody at 25 nmol/kg, IV and brains were collected following cardiac perfusion 18 h post injection (*n* = 2). Brain sections were stained for the presence of exogenous antibodies via IHC and images were collected from representative regions for comparison. Sections taken from the thalamus showed the distribution of the (**a**) PD-L1 antibody versus (**b**) TXB4-PD-L1. Prominent neuronal staining was only observed with the antibody fused to the shuttle. Pronounced TXB4-PD-L1 staining was seen in trigeminal neurons in the midbrain (**d**) and Purkinje cells in cerebellum (**f**), where diffuse parenchymal staining and BCEC staining were clearly distinguishable; PD-L1 antibody showed no staining in corresponding sections (**c**,**e**). Scale bars at 50 μm.

The ability of the TXB4 shuttle to transport a large therapeutic payload across the BBB to the brain parenchyma was confirmed using IHC (Figure 6). In brain sections of mice injected with TXB4-PD-L1 fusion construct at 25 nmol/kg dose and 18 h timepoint, prominent labeling was observed in BCECs and neurons throughout the brain, which was indistinguishable from that of the TXB4 shuttle itself in the same brain regions (see Figure 3). In stark contrast, the unmodified PD-L1 antibody did not label BCECs, neurons or the brain parenchyma (Figure 6) although staining in choroid plexus epithelial cells was observed—Supplementary Figure S1).

## 4. Discussion

As we have previously shown, efficient brain penetration can be achieved with the high affinity, bivalent VNAR shuttle to TfR1 called TXB2, which was isolated from semi-synthetic VNAR library by a combinatorial in vitro/in vivo selection [15]. TXB2 has a number of features that differentiate it from conventional IgG-based TfR1 antibodies. Namely, TXB2 exhibits species cross-reactivity and brain tissue selectivity, and penetrates the brain at low doses without depleting reticulocytes from the plasma or endogenous TfR1 from the BBB. Here, we describe the in vitro maturation of TXB2 by site-directed mutagenesis of the CDR3 region, which is predominately responsible for antigen binding [16,17].

The antigen binding region of TXB2 is unusual in that the cysteine in the CDR3, which forms a disulfide bridge with the CDR1 loop, occurs near the C-terminus at position 12 rather than in the middle. To preserve this disulfide bridge, only the 11 most N-terminal of the 14 total residues were randomized. Five sub-libraries were generated (L1–L5) containing three successive randomized residues and each consecutive sub-library contained one residue that overlapped with the previous library (Figure 1a). The sub-libraries were pooled and selected against hTfR1. The pre- and post-selection analysis of TfR1 binding showed the predominance of two sub-libraries (L3 and L4), which span residues in positions 5–9. Mutations to the CDR3 flanks abolished target binding unless substituted with biochemically similar residues. The high correlation in binding between hTfR1 and mTfR1 among the TXB2 variants indicate that the epitope recognition site is conserved cross-species. Although the exact residues that interact with TfR1 have not been identified, a simple explanation is that the loop flanks are critical in providing structural and spatial orientation to the middle CDR3 region that directly binds the epitope.

CDR3 randomization generated numerous TXB2 variants that retained TfR1 binding and 47 were tested for brain penetration in mice. Of these binders, 9 showed higher brain levels than TXB2, another 9 were approximately equivalent, while 14 had reduced brain levels and 15 lost the activity. These results indicate that TfR1 binding was necessary for but did not guarantee the brain penetration. Furthermore, the data suggest a more complex CDR3 structure–activity relationship whereby the N-terminal region binds the receptor while variations in the mid region enhance, are neutral or hinder brain penetration in vivo. This might occur through interactions with the dense glycocalyx coating the entire luminal surface of brain capillaries, which presents an additional physical and electrical barrier [24]. A more detailed analysis has revealed specific patterns and context of allowed substitutions in the mid region of CDR3 loop for further exploration [25].

To address whether differences in TfR1 binding kinetics affect functional activity in vivo, 19 TXB2 variants were selected across a range of brain penetration for SPR analysis. There was a strong positive and significant correlation between mTfR1 association rate (ka) and brain penetration in mice, whereas the dissociation rate (kd) was randomly distributed. While the correlation with affinity (KD) showed only a positive trend driven by the association rate, all the variants with enhanced brain penetration bound the receptor with high affinity (KD < 2 nM). VNAR shuttles with high association rate were shown to have an advantage in brain penetration where the velocity of blood flow may limit receptor exposure time followed by receptor engagement and transcytosis. Due to the complexity of the process, however, receptor association will be one of a multiplicity of contributing factors involved in the transport of TXB2 and its variants across the BBB.

The variant designated TXB4 was selected for further characterization since it had the highest TfR1 association rate and the highest level of brain penetration. The CDR3 modification did not result in epitope shift since TXB4 retained the unique brain specificity of TXB2 [15]. TXB4 did not accumulate in any of the tested organs except for the brain, where a 27-fold increase over control was observed. Since TXB4 did not target other organs, it is likely that lower plasma levels were a direct consequence of higher brain penetration, which was confirmed via IHC. A high level of specific immunoreactivity was observed across different brain regions in mice injected with TXB4 at a dose of only 25 nmol/kg (1.875 mg/kg). In addition to capillary staining, a strong reactivity was observed diffusely throughout the parenchyma and within neuronal cell bodies.

Using neurotensin (NT) as a payload, we previously found that TXB2 caused a significant drop in body temperature after IV injection [15]. Endogenous NT is expressed by glial cells and neurons, and upon binding to neurotensin receptor 1 (NTSR1) elicits several physiological effects including body temperature reduction [26]. Importantly, only brain expressed NTSR1 is directly linked to body temperature regulation and was previously utilized to confirm parenchymal penetration of other brain shuttles [15,22,27]. A TXB4-NT fusion dosed at 25 nmol/kg, IV, also reduced body temperature in mice. A significant reduction was observed from 1 to 5 h post injection with maximal effect at 2 h. The time course was similar to the parental TXB2 but the magnitude increased from approximately 2 °C to 2.9 °C, which most likely resulted from higher brain penetration. The gradual recovery of body temperature from the 2 h timepoint onwards was a consequence of receptor desensitization and not the brain levels, as was previously shown with TXB2 [15].

The ability of the TXB4 shuttle to deliver macromolecules to the brain was evaluated using various monoclonal antibodies dosed at 25 nmol/kg and measured at 18 h post injection. A bivalent N-terminal fusion of TXB4 to the heavy chain of a CD20 antibody (TXB4-CD20) increased brain penetration 20-fold over the unmodified antibody and nearly 3-fold improvement over the TXB2-CD20 fusion. Identical N-terminal bivalent fusions of EGFRvIII, PD-L1 and BACE1 antibodies with TXB4 were likewise compared to the unmodified antibodies and brain penetration increased from 14- to 30-fold. Consistently with other reports, the unmodified antibody showed percentage brain to plasma ratio ranging from 0.11 to 0.25% [13]. When fused to TXB4, the ratio increased and ranged between 4.9% and 6.2%, similar to previous reports and depside using an alternative low-affinity brain shuttle module in monovalent format at an approximately 5-fold higher dose [13]. A reduction in plasma levels was observable with the TXB4 shuttle fusions but only reached significance when fused to PD-L1. The plasma level reduction, which was not observed with TXB2 fusions, was attributed to the higher level of brain penetration provided by the TXB4 shuttle. IHC confirmed the brain parenchyma delivery of PD-L1 dosed at 25 nmol/kg, which localized not only in capillaries but also in the parenchyma and neuronal cell bodies. The pattern of TXB4-PD-L1 staining was very similar to TXB4 alone, which was expected since the PD-L1 antibody is human specific [21] and thus the tissue distribution was driven solely by TXB4. However, the biodistribution of a particular antibody–shuttle fusion will be influenced by the therapeutic target location and density, in addition to antibody binding affinity.

Aside from robust brain penetration of high affinity, bivalent formats, additional features distinguish VNAR-based from IgG-based TfR1 shuttles, which may stem from the unique structure of their antigen binding paratope [17]. TXB2 and its variants show a remarkable degree of brain selectivity despite the expression of TfR1 by cells in multiple organs [28]. By comparison, monoclonal antibodies to mouse TfR1, such as RI7217 and 8D3 specifically accumulate in heart, liver and kidney in addition to brain [29,30]. A smaller paratope provides access to a larger number of surfaces and buried epitopes, which may allow the discrimination between receptors with different post-translation modifications and in different microenvironments. TXB2 and its variants also show cross-species reactivity, which is difficult to obtain with IgG-based TfR1 antibodies. There is considerable divergence in TfR1 surface residues across species due to selective pressure by multiple viruses that uses the a receptor for cellular entry [31] and VNARs may more readily bind highly conserved cryptic epitopes inaccessible to conventional antibodies [16,32].

## 5. Conclusions

While the TXB2 VNAR shuttle showed efficient brain penetration at low therapeutic doses, its activity could be further improved by CDR3 mutagenesis. The TfR1 association rate in vitro directly correlated with activity in vivo where TXB4 showed the highest association rate and the highest brain levels. The bivalent TXB4 shuttle enabled a robust physiological response to NT peptide in the CNS and increased brain levels of therapeutic antibodies up to 30-fold. However, the range of therapeutic payloads extends beyond peptides and antibodies to enzymes, growth factors and anti-sense oligonucleotides. The unique features of the VNAR shuttle confer distinct advantages over alternative approaches. In addition to a lower effective dose range, the brain specificity improves efficacy and safety, and cross-species reactivity presents an opportunity for direct clinical translation.

## 6. Patents

Stocki, P., Wicher, K.B., Szary, J.M., and Rutkowski, J.L., inventors. Ossianix, Inc., assignee. Improved TfR-selective binding peptides capable of crossing the blood brain barrier. International Publication No. WO2019089395A1, published 9 May 2019.

Stocki, P., Wicher, K.B., Rutkowski, J.L., Comper, F., Demydchuk, M., and Szary, J.M., inventors. Ossianix, Inc., assignee. In vivo methods for selecting peptides that cross the blood brain barrier, related compositions and methods of use. International Publication No. WO2021102276A1, published 13 June 2019.

## Figures and Tables

**Table 1:** Binding kinetics of TXB2 and TXB4 to hTfR1 and mTfR1. To determine binding kinetic properties, a multicycle SPR analysis was performed using Biacore.

	hTfR1			mTfR1		
	ka (1/Ms)	kd (1/s)	KD (M)	ka (1/Ms)	kd (1/s)	KD (M)
**TXB2**	7.2 × 10^4^	5.2 × 10^−4^	7.2 × 10^−9^	2.5 × 10^5^	1.6 × 10^−4^	6.5 × 10^−10^
**TXB4**	2.2 × 10^5^	7.4 × 10^−4^	3.4 × 10^−9^	5.2 × 10^5^	2.9 × 10^−4^	5.5 × 10^−10^

## Data Availability

Not applicable.

## Supplementary Materials

The following supporting information can be downloaded at: https://www.mdpi.com/article/10.3390/pharmaceutics15030739/s1, Figure S1: IHC assessment of choroid plexus. (**a**) The untreaded control was obtained from non-injected animals and served as a staining control. Mice were dosed with either (**b**) the negative control VNAR-hFc (neg. cont.), (**c**) TXB4, (**d**) PD-L1 antibody or (**e**) TXB4-PD-L1 at 25 nmol/kg, IV and brains were collected following cardiac perfusion 18 h post injection (*n* = 2). Brain sections were analyzed for the presence of exogenous antibodies by IHC and images were collected from choroid plexus for comparison. 500 μm scale bar shown in (**a**).
