# Supplementary material for: CDR3 Variants of the TXB2 Shuttle with Increased TfR1 Association Rate and Enhanced Brain Penetration
Source: Pharmaceutics. 2023 Feb 23;15(3):739. doi: 10.3390/pharmaceutics15030739 (PMC10051654; doi:10.3390/pharmaceutics15030739)
Supplement: Supplementary file 1 [file pharmaceutics-15-00739-s001.zip › pharmaceutics-2144539-supplementary.pdf]

pharmaceutics

MDPI

Article

CDR3 Variants of the TXB2 Shuttle with Increased TfR1 Association Rate and Enhanced Brain Penetration

Pawel Stocki [1,†], Jaroslaw Szary [1,†], Mykhaylo Demydchuk [1], Leandra Northall [1], Charlotte L. M. Rasmussen [2], Diana Bahu Logan [1], Aziz Gauhar [1], Laura Thei [1], Shu-Fen Coker [1], Torben Moos [2], Frank S. Walsh [1] and J. Lynn Rutkowski [1,*]

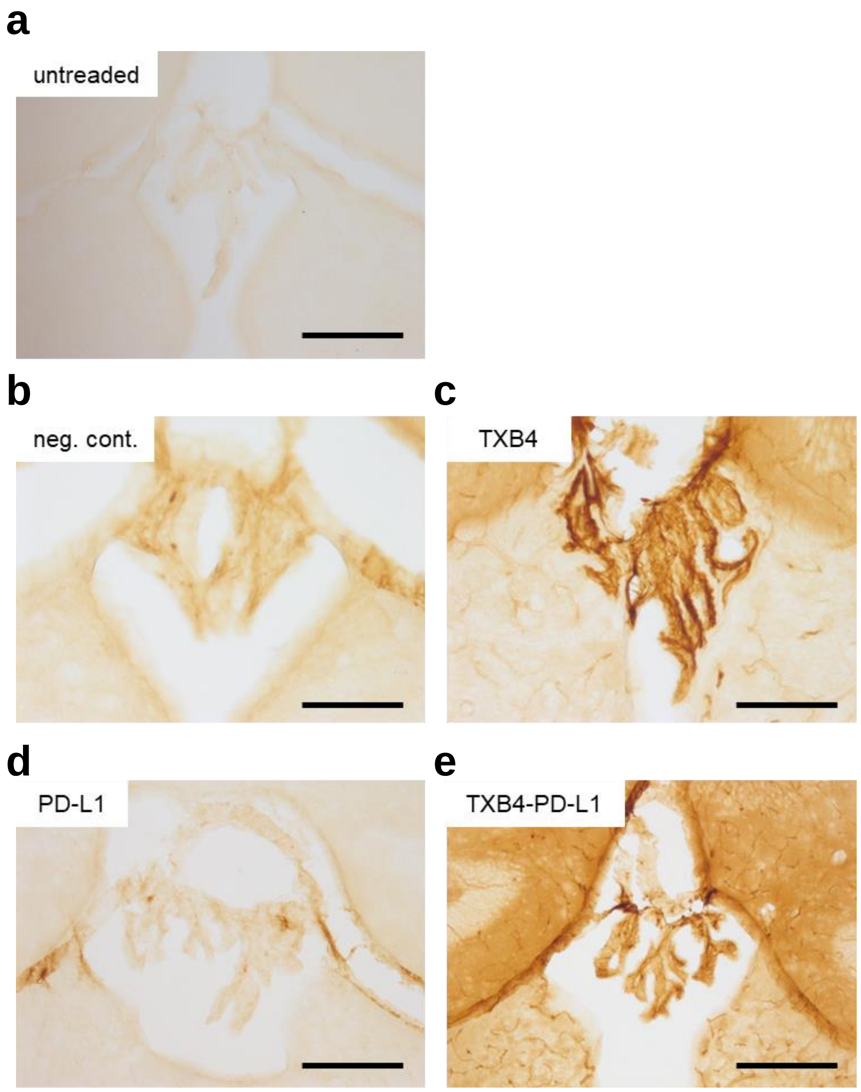

Supplementary Figure S1. IHC assessment of choroid plexus. **(a)** The untreaded control was obtained from non-injected animals and served as a staining control. Mice were dosed with either **(b)** the negative control VNAR-hFc (neg. cont.), **(c)** TXB4, **(d)** PD-L1 antibody or **(e)** TXB4-PD-L1 at 25 nmol/kg, IV and brains were collected following cardiac perfusion 18 hrs post injection (n = 2). Brain sections were analyzed for the presence of exogenous antibodies by IHC and images were collected from choroid plexus for comparison. Scale bars at 500 μm.
